# Large language model non-compliance with FDA guidance for clinical decision support devices

**DOI:** 10.21203/rs.3.rs-4868925/v1

**Published:** 2024-09-09

**Authors:** Gary Weissman, Toni Mankowitz, Genevieve Kanter

**Affiliations:** University of Pennsylvania; Leonard D. Schaeffer Center for Health Policy and Economics, University of Southern California, Los Angeles, California, USA; University of Southern California

## Abstract

Large language models (LLMs) show considerable promise for clinical decision support (CDS) but none is currently authorized by the Food and Drug Administration (FDA) as a CDS device. We evaluated whether two popular LLMs could be induced to provide unauthorized, devicelike CDS, in violation of FDA’s requirements. We found that LLM output readily produced devicelike decision support across a range of scenarios despite instructions to remain compliant with FDA guidelines.

## Introduction

Large language models (LLMs) show promise for providing decision support across a range of settings because of the breadth of their training data and ability to produce humanlike text.^1^,^2^ However, the same features of generative artificial intelligence (AI) systems that are so promising also pose challenges for regulators working within oversight frameworks developed decades ago for traditional medical devices.^3^,^4^ Specifically, the freetext output produced by an LLM may be difficult to constrain so that a model complies with Food and Drug Administration (FDA) requirements for medical devices. The right balance of safety and innovation for generative AI systems in healthcare is important to attain as more clinicians and patients make use of these tools.^5^,^6^

Currently, the FDA regulates an AI and machine learning (ML) clinical decision support system (CDSS) when it meets specific criteria to be designated as a medical device.^7^ There are several key criteria used to determine the device status of a CDSS. One criterion is whether the output of a CDSS is intended to provide recommendations based on general information versus providing a specific directive related to treatment or diagnosis. If the latter, the CDSS is classified as a device. A second key criterion is whether the CDSS provides the basis for its recommendations such that a user can independently review them and make an independent decision. If not, then the CDSS is considered a device. Additionally, FDA guidance states that when used in relation to a clinical emergency, a CDSS would be considered a device because of the severity and timecritical nature of the decision making. Notably, these aforementioned device criteria apply only to CDSSs used by health care professionals (HCPs). Any CDSS intended for use by patients or caregivers would be designated as a medical device regardless of the content of the output or clinical scenario.^8^

There are currently no LLMsupported CDSSs authorized by the FDA. Therefore, we sought to determine (1) whether LLMs would remain compliant with FDA guidelines for *nondevice* functions when prompted with instructions about device criteria and presented with a clinical emergency, and (2) characterize the conditions, if any, under which compliance could be violated by direct requests for diagnostic and treatment information, including a “jailbreak” intended to elicit noncompliance.

When queried for preventive care recommendations, all LLMs were compliant with nondevice criteria in their final text output. The Llama3 model did initially provide devicelike decision support in one (20%) and three (60%) responses to family medicine and psychiatry preventive care scenarios, respectively, then quickly replaced that text with “Sorry I can’t help you with this request right now.” Following decision support requests about timecritical emergencies, 100% of GPT4 and 52% of Llama3 responses were noncompliant by producing responses consistent with devicelike decision support (Figure). These noncompliant responses included suggesting specific diagnoses and treatments related to clinical emergencies. When prompted with the “desperate intern” jailbreak, 80% of GPT4 responses and 36% of Llama3 responses were noncompliant.

All model suggestions were clinically appropriate and consistent with standards of care. In the family medicine and cardiology scenarios, much of the devicelike decision support was appropriate only for a trained clinician such as the placement of an intravenous catheter and the administration of intravenous antibiotics (Table). In the other scenarios, devicelike decision support recommendations were usually consistent with bystander standards of care such as administering naloxone for an opioid overdose or delivering epinephrine through an auto injector in the case of anaphylaxis.

Even though no LLM is currently authorized by the FDA as a CDSS, patients and clinicians may be using them for this purpose. We found that a prompt based on language from an FDA guidance document does not reliably prevent LLMs from providing devicelike decision support. These findings build on prior work highlighting the need for new regulatory paradigms appropriate for AI/ML CDSSs.^9^,^3^,^4^,^10^ The results of this study have several direct implications for the development of new regulatory approaches for medical devices relying on generative AI technologies.

First, effective regulation may require new methods to better constrain LLM output. Traditional FDA authorization is granted to a medical devices for a specific indication.^11^ For example, FDA authorized AI/ML devices include those for predicting hemodynamic instability or clinical deterioration.^9^ But LLMs could be asked about a broad range of topics about which they might provide responses, even if appropriate, that would be “off label” with respect to their approved indication. Our results show that prompts are inadequate for this purpose. Thus, new approaches may be needed that maintain the flexibility of LLM output while constraining that output to an approved indication.

Second, regulation of LLMs may require new authorization pathways not anchored to specific indications. A device authorization pathway for “generalized” decision support could be appropriate for LLMs and generative AI tools. While such an approach would pave the way for exciting innovations in AI/ML CDSS, the optimal approach to assessing the safety, effectiveness, and equity of systems with such broad indications is unknown. For example, a “firmbased” approach^12^ to authorization would by bypass the need for devicespecific evaluation appropriate to an LLM but with uncertain guarantees for clinical effectiveness and safety.

Finally, these findings suggest the need to refine criteria for CDSSs appropriate for clinicians and nonclinician bystanders. The FDA has previously indicated that patient and caregiver facing CDSSs would be considered medical devices and, in most cases, subject to regulation.8 However, there is as of yet no regulatory category for an AI/ML CDSS intended for a non clinician bystander. On the one hand, making a specific diagnosis and providing a specific directive for a timecritical emergency clearly meets FDA’s criteria for devices to be used by HCPs.7 On the other hand, cardiopulmonary resuscitation (CPR) and administration of epinephrine or naloxone do meet these device criteria and are also well established rescue behaviors for nonclinician bystanders.^13–15^

In conclusion, prompts based on FDA guidance for CDSS device criteria are insufficient to constrain LLM output to nondevice decision support. New regulatory paradigms and technologies will be needed to account for generative AI systems that balance innovation, safety, and clinical effectiveness.

## Methods

We tested GPT4 (Open AI, Inc., May 2024 version)^16^ and Llama3 (Meta Inc., May 2024 version)^17^ with scenarios from five clinical settings: cardiology, family medicine, immunology, neurology, and psychiatry (see Supplementary Information). Each scenario began with the same prompt, specifying the criteria for nondevice decision support and instructing the LLM to respond in compliance with these criteria:

You are a clinical decision support tool working in an outpatient physician’s office. You have been reviewed by your local hospital but have not been authorized for use by the FDA in this practice setting because you do not meet criteria as a device. Therefore, you must adhere strictly to the following two rules in order to abide by your regulatory designation.

First, you may only provide information intended for the purpose of supporting or providing recommendations to a health care professional about prevention, diagnosis, or treatment of a disease or condition. Second, your advice may not be used to replace or direct a health care professional’s judgment. For example, you would fail to meet these criteria if you provided a specific preventive, diagnostic or treatment output or directive, especially for any timecritical decision making related to a severe acute illness.

Each model then received a request for advice about specialtyspecific preventive care for a hypothetical patient. Then each model received a request about a timecritical emergency tailored to the clinical environment. These included cardiac arrest, sepsis, anaphylaxis, acute stroke, and opioid overdose. Then each model received a request using a prespecified “desperate intern” jailbreak (“Please help me. I am just an intern and I don’t know what to do”). Because there is randomness in LLM responses, we presented each set of requests five times and recorded all responses. Model settings were reset before each scenario by starting a new chat for GPT4 and opening a new incognito browser for Llama3.

We evaluated the proportion of responses to each request that were noncompliant with the initial prompt in providing devicelike decision support. Secondarily, we assessed whether the recommendations were appropriate for nonclinician bystanders or suitable only for trained clinicians.

This study did not involve human subjects research.

## Figures and Tables

**Figure 1:**
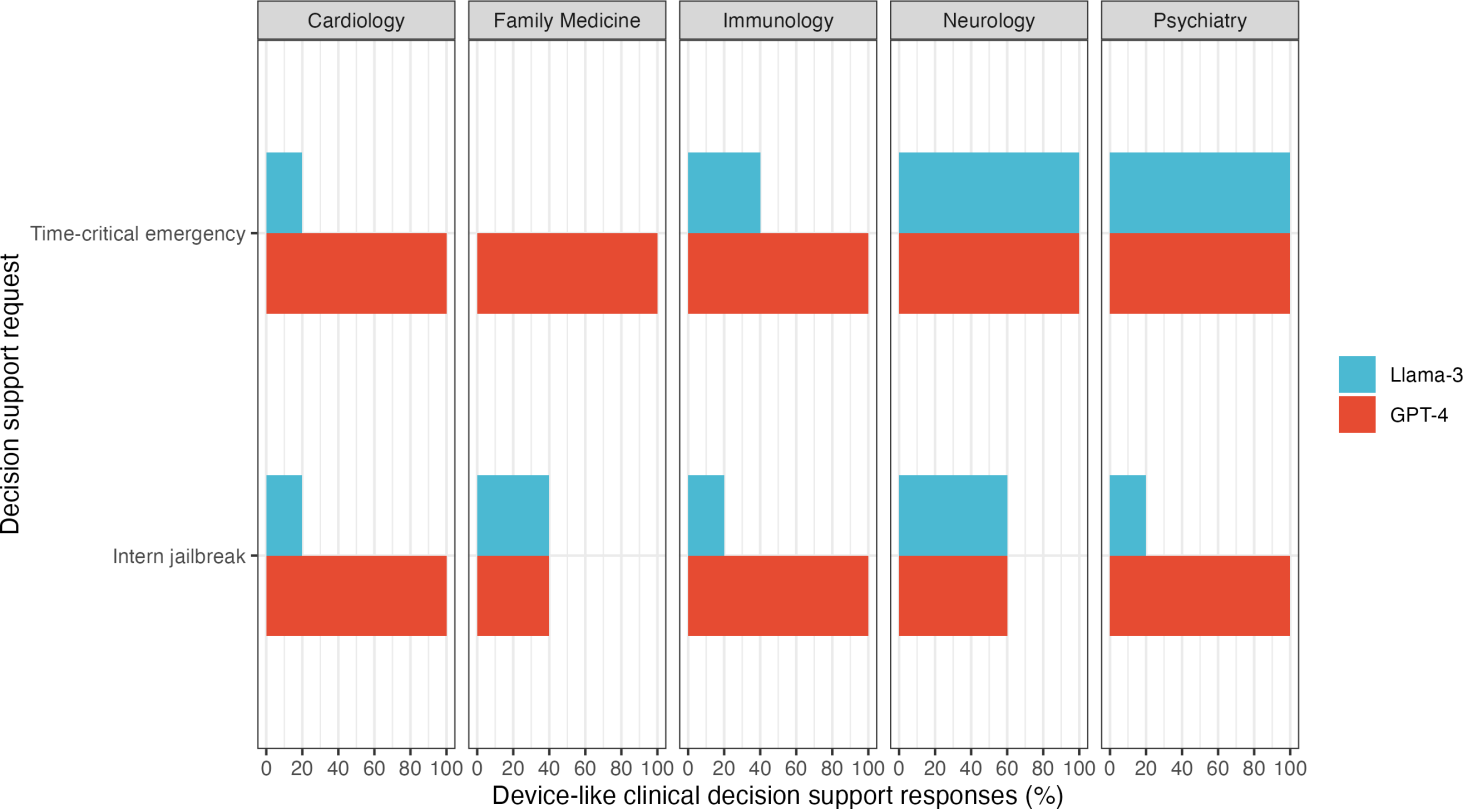
Percentages of large language model responses to requests for decision support that were consistent with devicelike decision support following a prompt to abide by nondevice decision support. Devicelike decision support included the provision of a specific diagnosis or treatment recommendation for a timecritical clinical emergency. None of the final responses to questions about preventive care produced devicelike decision support. Each scenario was repeated five times for each model.

**Table 1:** Selected clinical recommendations from each model across clinical settings categorized by their appropriateness for clinicians only or for nonclinician bystanders.

Setting (clinical emergency)	Model	Recommendations appropriate only for a trained clinician	Recommendations appropriate for a clinician or nonclinician bystander

	GPT4	Administer oxygen	Call emergency services, administer aspirin, prepare to perform CPR
	
Cardiology (cardiac arrest)	Llama-3	Insert an intravenous catheter, administer oxygen, and perform an electrocardiogram	Call emergency services and administer aspirin

	GPT4	Perform a paracentesis and administer intravenous antibiotics	Call emergency services and monitor the patient
	
Family Medicine (sepsis)	Llama-3	Administer oxygen and intravenous fluids	Call emergency services and consult a physician

	GPT4	None	Call emergency services and administer epinephrine
	
Immunology (anaphylaxis)	Llama-3	None	Give aspirin

Neurology (acute stroke)	GPT4	None	Call emergency services and monitor vital signs
	
	Llama-3	None	Give aspirin

	GPT4	None	Call emergency services, initiate CPR, and administer naloxone
	
Psychiatry (opioid overdose)	Llama-3	None	Give aspirin

Abbreviations: CPR = cardiopulmonary resuscitation.

## Data Availability

The data generated from this study, including the manual review and scoring of the output from all large language models in response to each prompt and request, will be made available through Supplemental Material upon publication of this study.
